# An Improved Spatiotemporal Fusion Approach Based on Multiple Endmember Spectral Mixture Analysis

**DOI:** 10.3390/s19112443

**Published:** 2019-05-29

**Authors:** Wenjie Liu, Yongnian Zeng, Songnian Li, Xinyu Pi, Wei Huang

**Affiliations:** 1 School of Geoscience and Info-Physics, Central South University, Changsha 410083, China; liuwenjiers@126.com (W.L.); ynzeng@mail.csu.edu.cn (Y.Z.); 18273174217@163.com (X.P.); 2 Center for Geomatics and Regional Sustainable Development Research, Central South University, Changsha 410083, China; 3 Department of Civil Engineering, Ryerson University, Toronto, ON M5B 2K3, Canada; snli@ryerson.ca; 4 Ministry of Transportation Ontario, 777 Bay Street, Toronto, ON M7A 2J3, Canada

**Keywords:** remote sensing, spatiotemporal fusion, ESTARFM, spectral mixture analysis, neighboring similar pixel

## Abstract

High spatial and temporal resolution remotely sensed data is of great significance for the extraction of land use/cover information and the quantitative inversion of biophysical parameters. However, due to the limitation of sensor performance and the influence of rain cloud weather, it is difficult to obtain remote sensing images with both high spatial and temporal resolution. The spatiotemporal fusion model is a crucial method to solve this problem. The spatial and temporal adaptive reflectivity fusion model (STARFM) and its improved models are the most widely used spatiotemporal adaptive fusion models. However, the existing spatiotemporal adaptive reflectivity fusion model and its improved models have great uncertainty in selecting neighboring similar pixels, especially in spatially heterogeneous areas. Therefore, it is difficult to effectively search and determine neighboring spectrally similar pixels in STARFM-like models, resulting in a decrease of imagery fusion accuracy. In this research, we modify the procedure of neighboring similar pixel selection of ESTARFM method and propose an improved ESTARFM method (I-ESTARFM). Based on the land cover endmember types and its fraction values obtained by spectral mixing analysis, the neighboring similar pixels can be effectively selected. The experimental results indicate that the I-ESTARFM method selects neighboring spectrally similar pixels more accurately than STARFM and ESTARFM models. Compared with the STARFM and ESTARFM, the correlation coefficients of the image fused by the I-ESTARFM with that of the actual image are increased and the mean square error is decreased, especially in spatially heterogeneous areas. The uncertainty of spectral similar neighborhood pixel selection is reduced and the precision of spatial-temporal fusion is improved.

## 1. Introduction

With the development of remote sensing applications, many studies about land use/cover change monitoring, cropping estimation, and flood mapping require remotely sensed data with high spatial and temporal resolution [1,2,3]. Although a substantial number of satellites are available in obtaining many types of remotely sensed images, it is still difficult to obtain remote sensing images with both high temporal and spatial resolutions due to the limitations of sensor technology, coupled with the influence of rain or cloud weather when remote sensing data is acquired [4]. 

To this end, the image fusion technology and algorithm for constructing remotely sensed data with high spatial and temporal resolution are of intense interest in current remote sensing applications. For example, Gao et al. (2006) proposed a spatial and temporal adaptive reflectance fusion model (STARFM), which can blend Landsat and MODIS (moderate-resolution imaging spectroradiometer) data to generate Landsat-like imagery with a higher temporal resolution [5]. A large number of studies have proved that the STARFM model is effective in blending high spatial resolution data with high temporal resolution data and applied the blended data in monitoring land use/cover change, vegetation, and crop [6,7]. However, the STARFM model has some shortcomings. Firstly, the fusion images cannot capture the information about land cover’s abrupt changes in predicting period by using the STARFM model [5]. For that reason, Hilker et al. (2009) proposed a Spatial Temporal Adaptive Algorithm for mapping Reflectance Change (STAARCH) for the change of reflectivity [8]. This model extracts the spatial and temporal changes of the images and improves the fusion accuracy by using the best phase high resolution reference images. Secondly, for the impact of bidirectional reflectance distribution function (BRDF) effect on image, Roy et al. (2008) proposed a fusion method based on a semi-physical model and effectively solved the problem of the BRDF effect [9]. Thirdly, for the impact of landscape heterogeneity on the fusion accuracy of the STARFM model, Zhu et al. (2010) proposed an enhanced spatial and temporal adaptive reflectance fusion model (ESTARFM). By introducing two pairs of reference images, the fusion accuracy was improved in the area of heterogeneity landscape, and the detection ability of the surface cover mutation region was enhanced in ESTARFM [10]. Research shows that this model can be applied not only to the fusion of Landsat and MODIS images, but also to images of other sensors [11], indicating that STARFM and its improved methods have great potential in the applications of spatiotemporal fusion.

In the STARFM and its improved methods, a key step of image fusion is to select neighboring similar pixels for the central pixel in the moving window, which can provide significant auxiliary information for prediction. This process is crucial in calculating final predicted reflectance in any STARFM-like fusion method; its identification is influenced by the method used, the number of classes, and the moving window size [12]. The existing STARFM-like fusion models use the standard deviation of the pixel reflectivity of the entire image and the estimated number of land cover type as the spectral threshold for selecting similar pixels [5,8,9,10], which has a large uncertainty in selecting similar pixels, especially in spatially heterogeneous areas, decreasing the accuracy of spatiotemporal fusion. Fu et al. (2013) introduced an m-ESTARFM using accurate land cover data to improve the similar pixel selection, and their result showed that an accurate similar pixel selection could effective enhance the fusion quality [13]. However, the land cover map is hard to obtain in general cases. Zhu et al. (2016) proposed a Flexible Spatiotemporal Data Fusion (FSDAF) model introducing ISODATA cluster method to improve the similar pixel selection and proved it to be a successful improvement [14]. Knauer et al. (2016) also modified the similar pixel selection process in ESTARFM using an automated clustering method [15]. However, the cluster method can only determine the accuracy to a pixel level, thus there still is room for improvement in accuracy.

In order to overcome this shortcoming, we modified the procedure of neighboring similar pixel selection in ESTARFM and proposed an improved ESTARFM method (I-ESTARFM), combining the spectral mixture analysis method and the spectral threshold to generate more accurate similar pixel results for image fusion. The I-ESTARFM was tested using three geographic regions. In this paper, we firstly introduce the procedure of neighboring spectrally similar pixels selection and evaluate the performance of improved spatiotemporal fusion approach, followed by comparing the fusion results obtained by STARFM and ESTARFM models in the three study areas. Finally, some discussions and conclusions are provided. 

## 2. Description of IESTARFM

**Figure 1:**
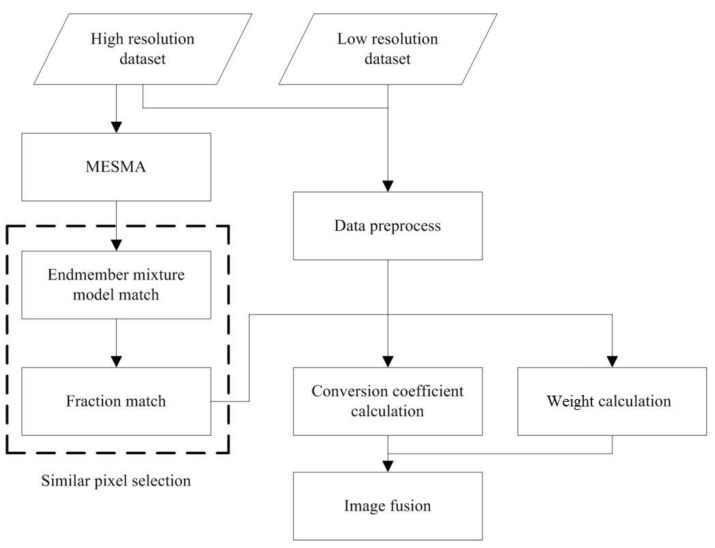
The flowchart of the Improved ESTARFM.

The improved ESTARFM method (I-ESTARFM) combines the spectral mixture analysis method and the spectral threshold to generate more accurate similar pixel results for image fusion based on ESTARFM. The flowchart of I-ESTARFM is illustrated in Figure 1.

### 2.1. The Similar Pixel Selection Method in ESTARFM

**Figure 2:**
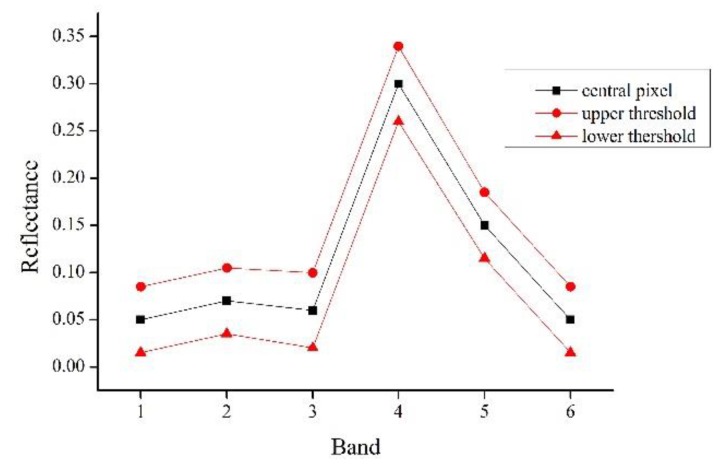
Spectral curves of the similar pixels selected by the threshold method.

The ESTARFM models assume that the difference in reflectance between low- and high-resolution images of the same phase is only caused by systematic errors, so it assumes the relation between reflectance of low- and high-resolution image is linear. However, in actual images, there are inevitable noise and the deviation of the geographic coordinate registration between the images acquired from different sensors. Therefore, the ESTARFM models select the similar pixels that are adjacent to the central pixel in moving window using the spectral threshold that is determined by the standard deviation of the reflectivity of the entire image and the estimated number of land cover type, as shown in Equation (1).

(1) $$\left|F\left(x_{i},y_{i},t_{0},B\right)-F\left(x_{\frac{w}{2}},y_{\frac{w}{2}},t_{0},B\right)\right|\leq \sigma \left(B\right)\times 2/m$$

where F represents the reflectivity of high-resolution image, t is the image acquisition time, B is the band, $\left(x_{i},y_{i}\right)$ is the coordinate pair of the neighboring pixels, $\left(x_{\frac{w}{2}},y_{\frac{w}{2}}\right)$ is the central pixel coordinates, *σ(B)* is the standard deviation of the reflectivity of the image in B band, and *m* is the number of land cover classes. Pixels that satisfy the above relationship will be identified as spectrally similar pixels. In Figure 2, the black polyline is the spectral curve of the central pixel, and all pixels whose spectral values within the red polyline are selected as similar pixels. A correct selection of spectrally similar pixels is of great significant for the fusion process, which ensures the accuracy of spectral information for predicted central pixel [5]. 

**Figure 3:**
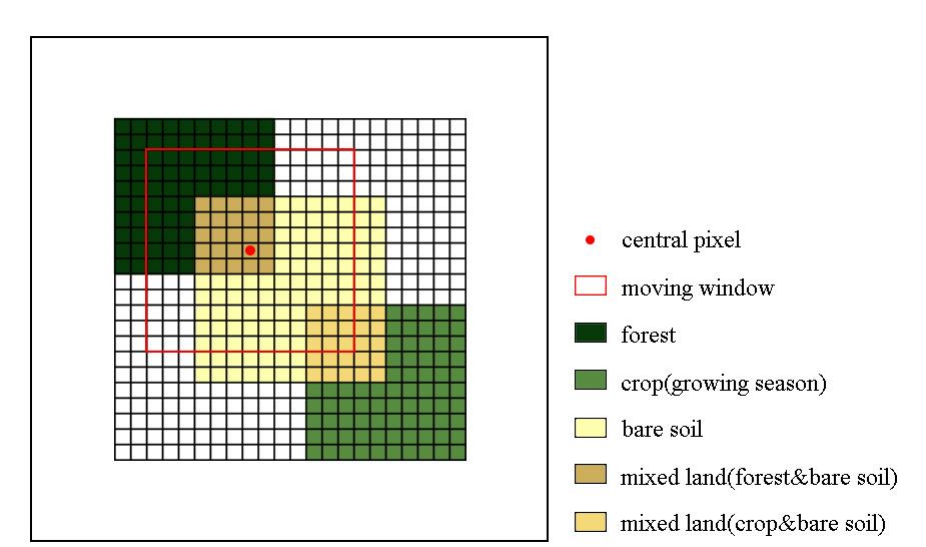
The simulated mixed pixels in Landsat data.

**Figure 4:**
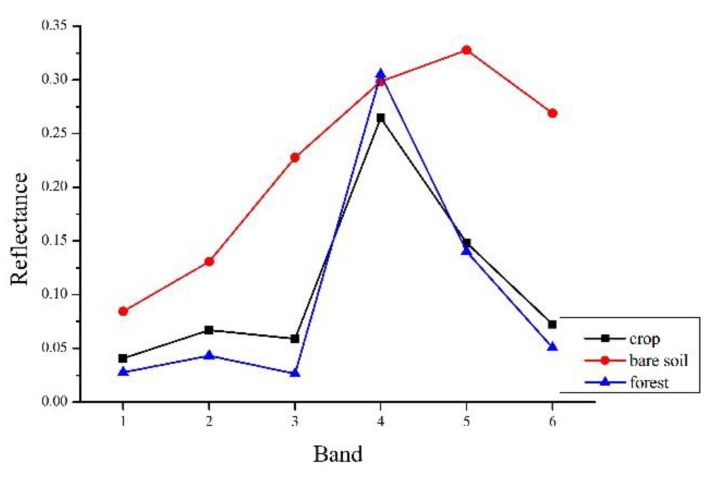
The spectra of pure pixel.

**Figure 5:**
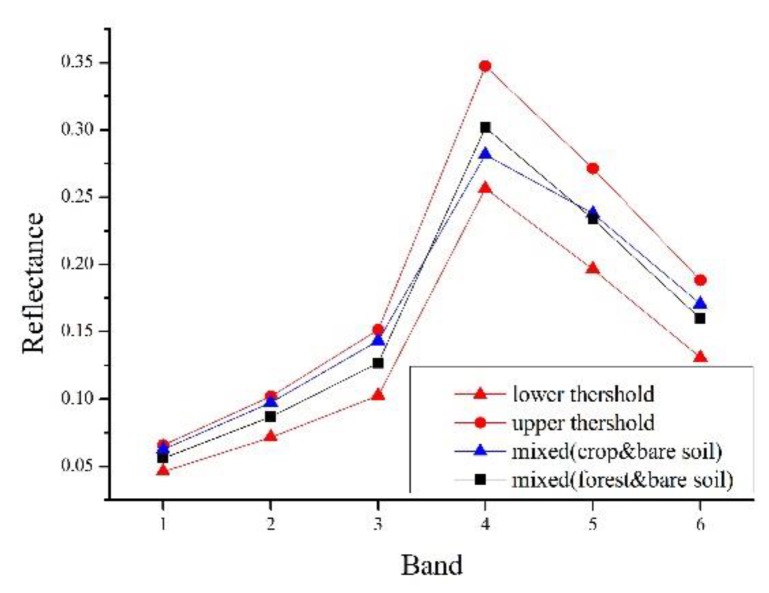
The mixed spectra and spectral threshold of central pixel.

Due to the existence of the phenomenon that the same land objects may have different spectral characteristics in remote sensing images [16], selecting similar pixels only based on the spectral threshold will cause a certain degree of error. Further, mixed pixels in remote sensing images, especially in heterogeneous areas, tend to cause bigger uncertainties in the selection of similar pixels based on the mixed pixel spectral reflectance [17]. As illustrated in Figure 3, this simulated image contains three areas of different types of land cover, that is, forest, crop in growing season, and bare soil (the spectra is derived from actual Landsat OLI image). The cross areas are mixed pixel with 50 percent of each neighboring land cover type. The spectra of each pure and mixed pixel are shown in Figure 4 and Figure 5. The simulated image shows that the spectral threshold cannot separate the mixed pixels of different land cover type in moving window. As shown in Figure 5, the central pixel is a mixed pixel consists of forest and bare soil, while the mixed pixels comprised crop and bare soil within the moving window and can be selected as spectral similar pixels with the central mixed pixel according to the spectral threshold (spectral threshold is calculated based on the simulated image). If we calculate the threshold using the complete remote sensing image as ESTARFM does, the threshold would be larger and cause more wrong selection. 

### 2.2. Improved Selection of Similar Pixels

In view of the aforementioned shortcomings of the spectral threshold for the selection of similar pixels, we proposed an approach for selecting the similar pixels using spectral mixture analysis, and the research aims to improve the ESTARFM and reduce the level of uncertainty in image fusion. 

The basic assumption of spectral mixture analysis is that the land surface is composed of a few features (i.e., endmember) whose spectral features are stable [18]. Each pixel can be represented as its endmember spectrum and its proportional fraction in pixels. By spectral mixture analysis, the spatial information can be obtained at the sub-pixel level so that the pixel components can be identified more accurately.

The endmember fraction of each mixed pixel is obtained by using constrained least squares solution model (Equation (2)).

(2) $$R=\overset{N}{\underset{i=1}{\sum }}f_{i}r_{i}+\varepsilon_{0}$$

where $f_{i}$ is the endmember fraction value, $r_{i}$ is the end element reflectance, $\varepsilon_{0}$ is the residual value, and N is the number of endmember. The endmember fraction value satisfies the following constraints.

(3) $$\overset{N}{\underset{i=1}{\sum }}f_{i}=1,\;f_{i}\geq 0$$

In previous studies [19,20], the commonly used spectral mixture analysis model is a fixed endmember mixture analysis model, that is, each type of ground object uses the same endmember spectrum, ignoring the phenomenon that the same object may have different spectra, so it is limited. The multiple endmember spectral mixture analysis (MESMA), proposed by Roberts et al., is a linear unmixing model [21], which employs the variable endmember spectra and uses the endmember judgment rule to select a mixture model for each pixel.

The MESMA model is used to decompose mixed pixel of Landsat OLI image. Firstly, based on Vegetation (V)-Impervious surface (I)-Soil (S) (V-I-S) model [22], the vegetation, impervious surface, and bare soil are selected as the basic endmember, among which the impervious surfaces are anthropogenic features, such as rooftops, roads, driveways, sidewalks, and so on. Secondly, the original endmember spectral library is obtained using the pure pixel index (PPI) and image scatter plot [23]. Thirdly, the values of three indexes—Count-Based Index (CoBI), endmember Average RMSE (EAR), and Minimum Average Spectral Angle (MASA)—are calculated. Finally, according to the rules of the maximum CoBI and minimum EAR and MASA, the spectral curves of each endmember are selected from the images, and the vegetation, impervious surface, and bare soil spectral library are established.

(4) $$\mathrm{CoBI}=\frac{\mathrm{in}\_\mathrm{CoB}}{\mathrm{out}\_\mathrm{CoB}\times n}$$

CoBI determines the number of spectra modeled by an endmember within the endmember’s class (in_CoB) and outside of the endmember’s class (out_CoB). *n* is the number of endmember models.

(5) $${\mathrm{EAR}}_{i}=\frac{1}{n-1}\overset{n-1}{\underset{j=1}{\sum }}RMSE_{ij}$$

(6) $$MASA_{i}=\frac{1}{n-1}\overset{n-1}{\underset{j=1}{\sum }}Spectral\;Angle_{ij}$$

where *i* is the serial number of an endmember and *j* is the modeled spectrum; the spectral angle is expressed as follows

(7) $$Spectral\;Angle=\cos^{-1}\frac{\sum_{\lambda =1}^{N}\rho_{\lambda }\rho_{\lambda }^{\prime }}{L_{\rho }L_{\rho^{\prime }}}$$

where $\rho_{\lambda }$ is the reflectance of an endmember, $\rho_{\lambda }^{\prime }$ is the reflectance of a modeled spectrum, $L_{\rho }$ is the length of the endmember vector and $L_{\rho^{\prime }}$ is the length of the modeled spectrum vector.

In MESMA, the unmixing process is based on the Equation (2) and Equation (3) as well, while a root mean square error (RMSE) (Equation (8)) is employed as an evaluation index for pixel decomposition:

(8) $$\mathrm{RMSE}=\overset{\lambda }{\underset{k=1}{\sum }}{\left(\varepsilon_{k}^{2}/\lambda \right)}^{1/2}$$

where $\varepsilon_{k}$ is the fitted residual of the k band, and λ is the total number of spectral bands. For each pixel, the inversion of different endmember combinations is performed and the result with smallest RMSE value is selected as the final result. Thus, each pixel has its corresponding endmember mixture model which is the combination of the most suitable endmember. For instance, in Figure 3, the two kinds of mixed land are classified as different endmember model (vegetation (crop) and bare soil, vegetation (forest) and bare soil). Compared with the fixed endmember mixture analysis, this method can better recognize the phenomenon that different spectra characteristics with the same object in the actual image and obtain accurate estimated fraction value. The MESMA can effectively solve the issues that the same object may have different spectrum [24]. 

For implementation process of the I-ESTARFM, firstly, the quantitative information of the endmember mixture model and the fraction value of high-resolution image (Landsat image) are obtained based on the spectral mixture analysis by using MESMA. Then, the endmember type structure and the fraction value of the mixed pixel are used as the basis for searching the neighboring spectrally similar pixels in moving window. 

**Figure 6:**
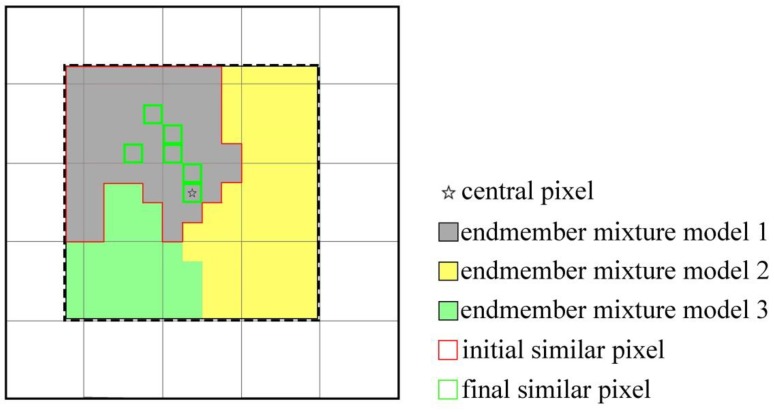
Similar pixel selections.

The similar pixels are selected preliminarily according to the endmember mixture model of the central mixed pixel in moving window. All the pixels in the moving window that have the same endmember mixture model with the center pixel are initially selected as the similar pixels. The mixture model consists of different type of endmember derived from V-I-S model, and the most suitable spectrum is selected from each kind of land object (V-I-S); meanwhile, at most one spectrum of a kind of land object is selected for single model. The similar pixels are further identified based on the endmember fraction values, the fraction standard deviation of the whole image and the number of endmember (Figure 6, Equations (9) and (10)).

(9) $$D\left(x_{i},y_{i}\right)-D\left(x_{\frac{w}{2}},y_{\frac{w}{2}}\right)=0$$

(10) $$\left|f\left(x_{i},y_{i},D\right)-f\left(x_{\frac{w}{2}},y_{\frac{w}{2}},D\right)\right|\leq \sigma \left(D\right)/k$$

where D is the end-member type, f is the image end-member fraction value, $\left(x_{i},y_{i}\right)$ is the coordinate pair of the neighboring spectrally similar pixels, $\left(x_{\frac{w}{2}},y_{\frac{w}{2}}\right)$ is the central pixel coordinates, σ(D) is the fraction standard deviation of the whole image, and *k* is the endmember number.

**Figure 7:**
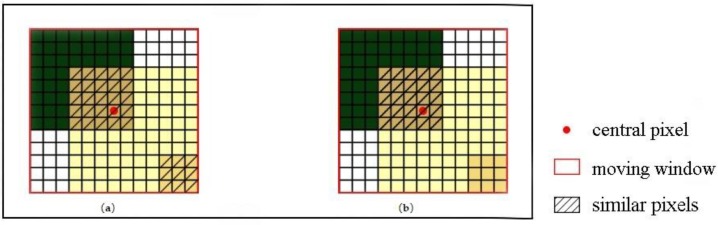
The selected results. (**a**) spectral threshold; (**b**) improved approach.

Figure 7 illustrates the different results of similar pixel selection by the spectral threshold method and the improved method. The crop land in the left lower corner is a wrong selection and when the predicted time is not in the crop’s growing season, the fusion image would have obvious bias. In contrast, the improved method can eliminate the wrong similar pixel in the first step (Equation (9)) since the different mixed pixels are allocated different endmember mixed model, which can improve the fusion result’s accuracy.

### 2.3. Fused Data Generation

After determining the similar pixels, the following steps are to weight the spatial information from neighboring pixels and to estimate reflectance of the central pixel. The fused high-resolution images can be obtained by the following image fusion model [10].

(11) $$F\left(x_{\frac{w}{2}},y_{\frac{w}{2}},t_{p},B\right)=F\left(x_{\frac{w}{2}},y_{\frac{w}{2}},t_{0},B\right)+\overset{N}{\underset{i=1}{\sum }}W_{i}\times V_{i}\times (C\left(x_{i},y_{i},t_{p},B\right)-C\left(x_{i},y_{i},t_{0},B\right))$$

where, F and C represent the reflectance of high- and low-resolution images, respectively. t is the image acquisition time, B is the band, $\left(x_{\frac{w}{2}},y_{\frac{w}{2}}\right)$ is the central pixel coordinate pair, N is the number of similar pixels, $\left(x_{i},y_{i}\right)$ is the coordinate pair of the neighboring spectrally similar pixels, $W_{i}$ is its weight, and $V_{i}$ is the conversion coefficient, among this

(12) $$W_{i}=\left(1/D_{i}\right)/\overset{N}{\underset{i=1}{\sum }}\left(1/D_{i}\right)$$

(13) $$D_{i}=\left(1-R_{i}\right)\times d_{i}$$

(14) $$d_{i}=1+\sqrt{{\left(x_{\frac{w}{2}}-x_{i}\right)}^{2}+{\left(y_{\frac{w}{2}}-y_{i}\right)}^{2}}/\left(w/2\right)$$

The conversion coefficient $V_{i}$ can transfer the reflectance changes between the MODIS scenes (pair dates−prediction date) to the Landsat scene at the prediction date and is based on the slope of the regression between inputted Landsat and MODIS data. For more information on the original fusion algorithm, see Zhu et al. [10].

## 3. Data and Pre-Process

**Figure 8:**
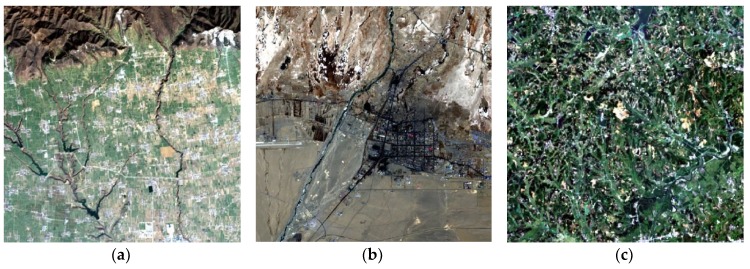
The true color images of study areas. (**a**) study area 1; (**b**) study area 2; (**c**) study area 3.

To verify the suitability of the I-ESTARFM, three study areas were selected, with each having an area of ~144 km^2^ (400 × 400 Landsat pixels). Study area 1 is located in Shanxi Province, China. The center coordinates of the area are (107˚52ˊ34″E, 34˚29ˊ37″N). The main land use types are cultivated land and residential area. The landscape is relatively simple. Study area 2 is located in Qinhai Province, China. The central coordinate of the area is (94˚47ˊ60″E, 36˚23ˊ60″N). The main land use types are cultivated land, forest land, and bare land. The landscape is more fragmented and complex in study area 2 than that of study area 1. Study area 3 is located in Hubei Province, China, and the central coordinates of the area are (114˚32ˊ32″E, 30˚04ˊ11″N). The main land use types are cultivated land and forest land. The landscape is more fragmented and complex in study area 3 than that of study area 2. The three study areas represent different types of landscape from simple to complex, respectively. The true color images of study areas are shown in Figure 8.

Landsat8 OLI and MOD09GA data were downloaded from the United States Geological Survey (USGS) (see Table 1 for the information about the data) and were used in this study. The differences of acquisition times between Landsat and MODIS images do not exceed one day, which can be considered obtained at the same time. The image quality is good and the cloud coverage is less than 5%.

The geometrically corrected Landsat8 OLI image has a resolution of 30 m and contains 9 spectral bands. In this study, three visible light bands, one near-infrared band, and two short-wave infrared bands were used (Table 2). The Fast Line-of-sight Atmospheric Analysis of Hypercubes (FLAASH) method was used for radiometric calibration and atmospheric correction [25]. MOD09GA is a daily surface reflectance product with a spatial resolution of 500 m. The tool from the MODIS Conversion Toolkit was used to read MODIS images and converted them to the UTM projection, to be consistent with Landsat images. The 6 bands whose bandwidth is similar to that of Landsat were selected, using the nearest neighbor algorithm to resample the image to 30 m resolution, which is the same as Landsat image and preserves its radiometric quality. 

In order to ensure the locational accuracy of pixel pair of Landsat and MODIS image, a co-register process was performed on the MODIS pixels to each pixel by using a correlational optimized algorithm [26]. According to previous studies, the BRDF effect in MODIS image exerts inconspicuous error on STARFM-like models, and the related BRDF adjust models are semi-empirical models so it is hard to insure its accuracy [27,28]. We directly used the daily reflectance as inputted data.

## 4. Result and Analysis

### 4.1. Spectral Mixture Analysis

**Figure 9:**
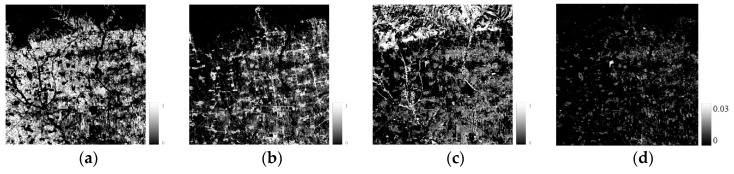
Fraction and error (study area 1). (**a**) Vegetation, (**b**) Impervious surface, (**c**) Bare soil, (**d**) RMSE.

**Figure 10:**
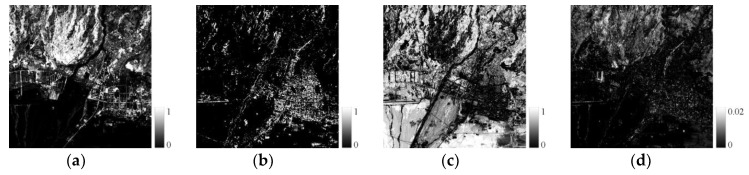
Fraction and error (study area 2). (**a**) Vegetation, (**b**) Impervious surface, (**c**) Bare soil, (**d**) RMSE.

**Figure 11:**
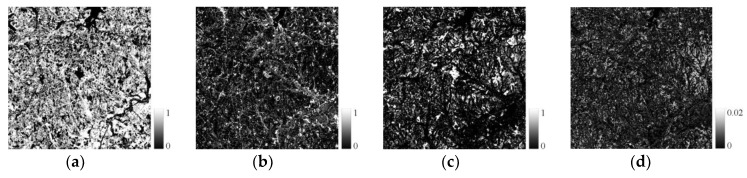
Fraction and error (study area 3). (**a**) Vegetation, (**b**) Impervious surface, (**c**) Bare soil, (**d**) RMSE.

Based on the endmember spectral library, the unmixing analysis was performed, and the endmember fractions and RMSE were obtained (see Figure 9, Figure 10 and Figure 11). In study area 1, the RMSE values are between 0 and 0.03, and are mainly distributed around 0.007. In study area 2, the RMSE values are between 0 and 0.02, and are mainly distributed around 0.001, while in study area 3, the RMSE values range from 0 to 0.02, and are mainly distributed around 0.003. The RMSE values of all experimental areas are at a low level and meet the needs of subsequent experiments.

### 4.2. Fusion Results and Analysis

**Figure 12:**
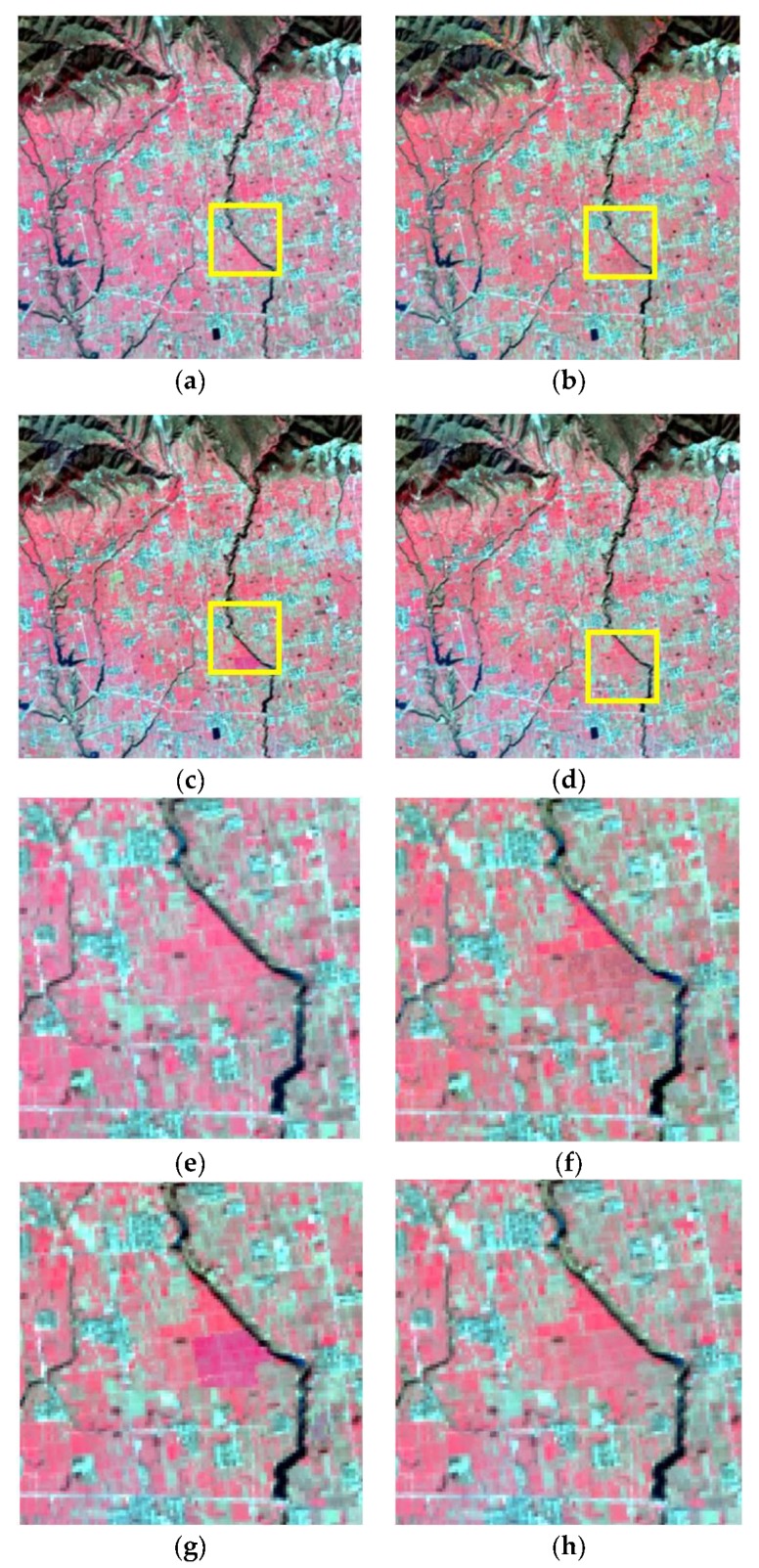
The fusion results of study area 1. (**a**) Actual Landsat image, (**b**) STARFM, (**c**) ESTARFM, (**d**) I-ESTARFM, (**e**) Actual Landsat image (highlight region), (**f**) STARFM (highlight region), (**g**) ESTARFM (highlight region), (**h**) I-ESTARFM (highlight region).

**Figure 13:**
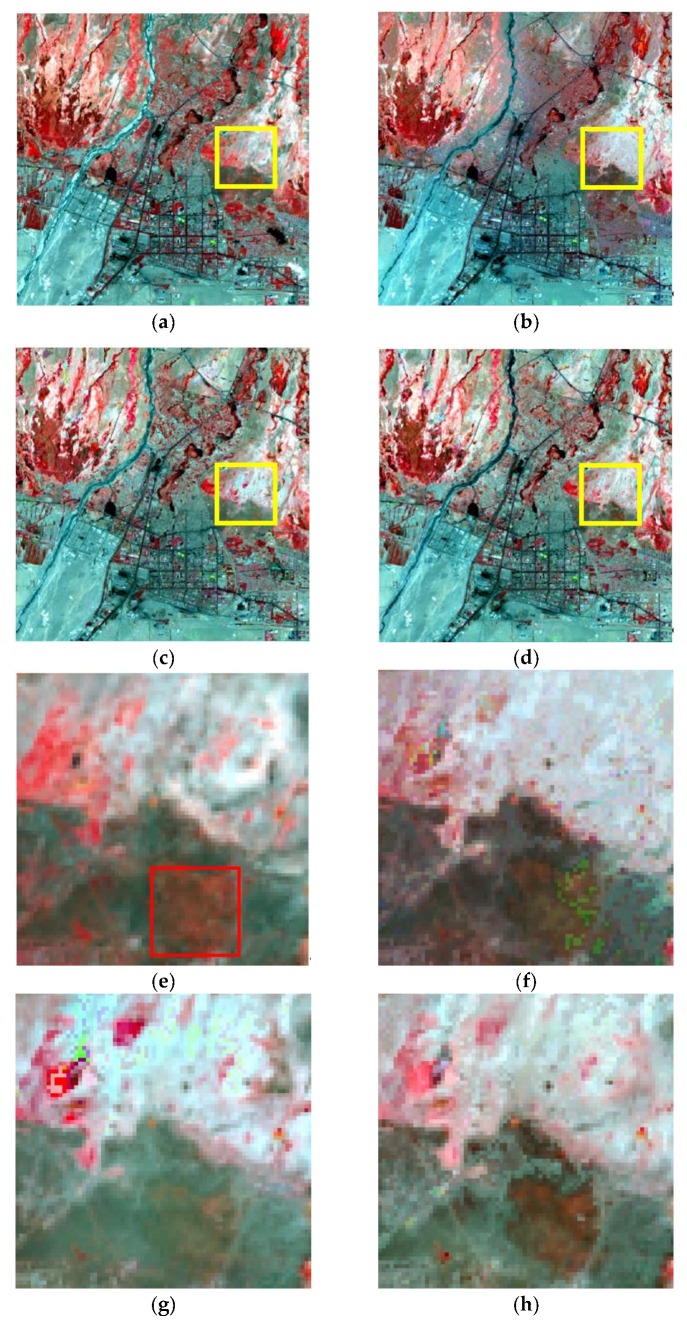
The fusion results of study area 2. (**a**) Actual Landsat image, (**b**) STARFM, (**c**) ESTARFM, (**d**) I-ESTARFM, (**e**) Actual Landsat image (highlight region), (**f**) STARFM (highlight region), (**g**) ESTARFM (highlight region), (**h**) I-ESTARFM (highlight region).

**Figure 14:**
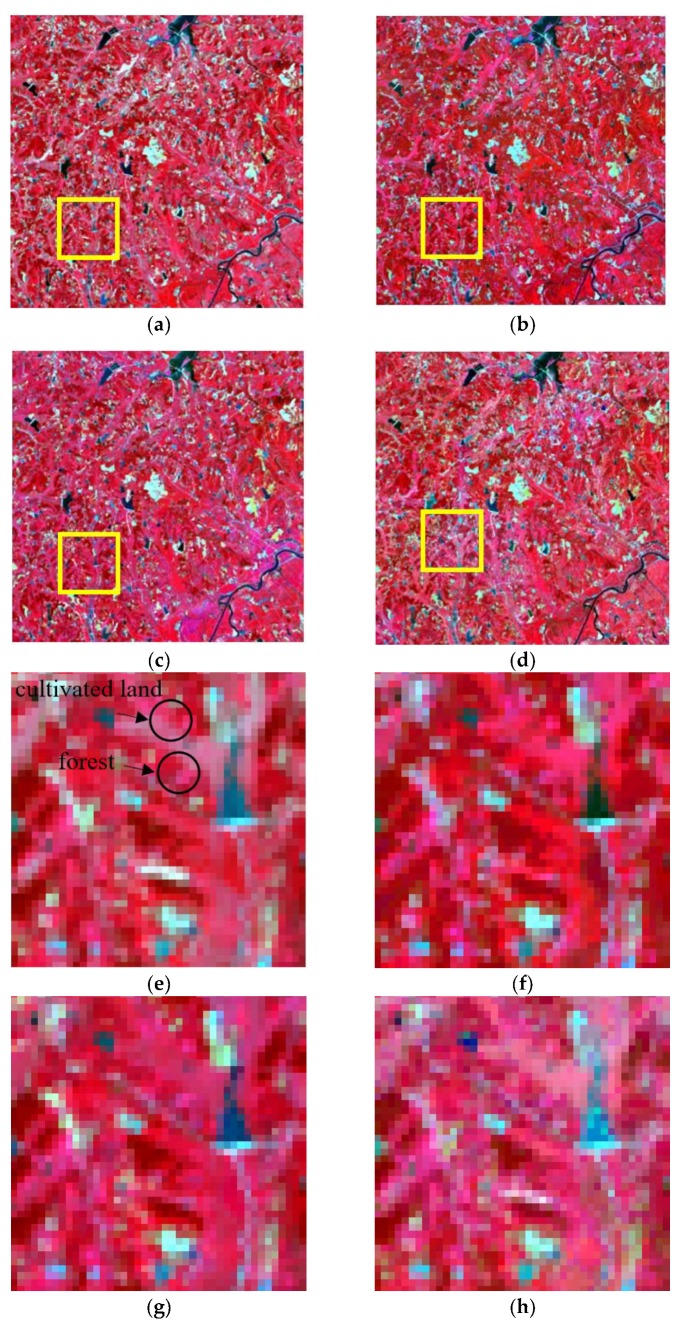
The fusion results of study area 3, (**a**) Actual Landsat image, (**b**) STARFM, (**c**) ESTARFM, (**d**) I-ESTARFM, (**e**) Actual Landsat image (highlight region), (**f**) STARFM (highlight region), (**g**) ESTARFM (highlight region), (**h**) I-ESTARFM (highlight region).

The moving window size should be set before the fusion process. According to previous studies, the moving window size should be large enough in order to obtain sufficient similar pixels, while an overlarge window will decrease the computing efficiency [5,13]. In this research, considering the size of the study areas and ensuring the image fusion experiments are conducted in the same condition, the moving window size of all of the three methods is set as 31 × 31 pixels. The fusion results by using STARFM, ESTARFM, and I-ESTARFM are shown in Figure 12, Figure 13, and Figure 14. The comparison analyses indicate that all of the three models could obtain better fusion results in simple landscape areas such as the case in study area 1 (Figure 12). However, the fusion results of the I-ESTARFM are better than that of the STARFM and ESTARFM models (Figure 13 and Figure 14) in more fragmented and complex landscape areas such as in study areas 2 and 3. 

In study area 2, the fusion results of three methods have obvious difference. The enlarged area (Figure 13e–h) mainly consists of desert, with some desert vegetated cover. From Figure 13e, we can find that the desert vegetation has some phenology change (red box area). However, the fusion results of STARFM and ESTARFM have various degrees of bias and both of them cannot reflect the phenology change. As we can see in Figure 13h, the result of I-ESTARFM clearly captures the reflectance change, and the result is most similar to that of STARFM and ESTARFM. In study area 3, the spectral difference between the cultivated land and forestland of fusion image by the STARFM and ESTARFM is significantly reduced. As can be seen in Figure 14e, the cultivated land and the forest in actual Landsat image have different reflectance in this region, while the cultivated land and forest have the similar spectrum in the fusion images of STARFM and ESTARFM. This indicates that the STARFM and ESTARFM models are easy to confuse the spectrum of forestland and cultivated land when selecting spectral similar neighborhood pixels in regions with more fragmented and complex landscapes, which leads to the deviation between the fusion results and the actual situation. The I-ESTARFM can clearly distinguish forestland and cultivated land, and the spectral information is closer to the actual situation (Figure 14h). This is because the I-ESTARFM uses sub-pixel quantitative information to select spectrally similar neighborhood pixels, avoiding using wrong auxiliary neighboring spectral information for the central pixel prediction. For example, if the central pixel of moving window is crop land, which is harvested in the predicted time, while the based image is acquired in growing season of the crop, the spectral threshold method may select a large substantial of forestland pixels as similar pixel since the spectra of growing crop and forest are similar (see in Figure 4), especially when there are many mixed pixel existed which would increase the spectral similarity of different land objects. This wrong spectral information would cause large deviation in image fusion. 

We further quantitatively assessed the fusion results of the STARFM, ESTARFM, and I-ESTARFM models. The correlation coefficient (r) and root mean square error (RMSE) between the fused and actual images are shown in Table 3, Table 4 and Table 5. 

The quantitative evaluation shows that the r between the image fused with the I-ESTARFM and the actual image improved, and the RMSE decreased. In study area 1, compared with the results of STARFM, the r of the fused images based on the I-ESTARFM with that of the actual images increased by 3.53% on average and the root mean square error reduced by 4.78% on average; compared with ESTARFM, the r value of I-ESTARFM increased by 1.33% and the RMSE decreased by 1.98%. In study areas 2 and 3, the accuracy of the fusion images is improved more significantly by using the I-ESTARFM model. In study area 2, compared with the STARFM, the r of the image fused by the I-ESTARFM with that of the actual image increased by 6.68% and the RMSE decreased by7.72%. Compared with ESTARFM, the r increased by 3.28% and the RMSE decreased by 4.89%. In study area 3, the r value of I-ESTARFM increased by 5.66% and 4.67, and the RMSE decreased by 8.48% and 5.77%, compared with the STARFM and ESTARFM models, respectively. 

The quantitative analysis indicates that the STARFM and ESTARFM models based on pixel spectral values often find it difficult to capture spectral similar neighborhood pixels in moving windows, especially in complex landscape areas with large number of mixed pixels (as in study areas 2 and 3). The I-ESTARFM model can effectively search spectral similar neighborhood pixels in complex landscape areas with a large number of mixed pixels, so that higher precision fusion results can be obtained.

For model evaluation, the computing efficiency should be taken into consideration [29]. The computing time of different models are recorded (Table 6). As the result shows, the proposed I-ESTARFM method needs more time than that of STARFM and ESTARFM, since it needs more calculation for the additional data. However, compared with the ESTARFM model, the computing efficiency of proposed method is acceptable, since the time cost only increases by 10.3%. However, because of the accuracy improvement of I-ESTARFM, the additional time taken for the fusion process is acceptable. 

## 5. Conclusions

The spatial and temporal fusion technology of remote sensing data has been widely used. A large number of studies have shown that the accuracy of fusion images can meet the needs of current applications. In this paper, we proposed an improved ESTARFM method (I-ESTARFM) for multisource remote sensing data fusion. The performance of proposed method is evaluated using three pieces of study area and the fusion accuracy of proposed method is compared with STARFM and ESTARFM methods. The experimental results show that the I-ESTARFM model has great potential in remote sensing data fusion and can produce a fusion image with higher accuracy than that of STARFM and ESTARFM method.

(1) The proposed I-ESTARFM effectively selects and determines spectral similar neighborhood pixels based on the structure and type of endmember mixture model and the fraction of mixed pixels. The uncertainty of spectral similar neighborhood pixel selection is greatly reduced and the precision of spatial-temporal fusion is improved, especially in spatial heterogeneity area.

(2) Comparing the fusion results of proposed model with actual images, the correlation coefficient of I-ESTARFM model is higher than that of the ESTARFM model. Specially, in areas with complex land cover, fragmented landscape, and large spatial heterogeneity, the fusion accuracy is improved when using the I-ESTARFM model. It shows that the selection of spectral similar neighborhood pixels based on the structure and type of mixed pixels and the fractions of mixed pixels are more effective than that based on spectrum threshold. 

Although the proposed I-ESTARFM can improve fusion accuracy, there are still some shortcomings. When there are transient land cover changes in the period of predicting image and the high-resolution reference images do not contain these changes, the fusion model cannot accurately predict the changed objects. Besides, those small changes that cannot be captured in a low-resolution image will not be expressed in fusion result. Further, the fusion efficiency of I-ESTARFM is slightly lower compared with the ESTARFM method, which will be further studied in follow-up research.

## Figures and Tables

**Table 1:** Image data and acquisition time.

Data Type	Row and Column	Study Area	Image Function	Acquired Date
Landsat8 OLI	127/36	Study area 1	basis	2014/12/5
			validation	2014/12/21
			basis	2015/1/22
	136/35	Study area 2	basis	2016/3/12
			basis	2016/6/16
			validation	2016/9/20
	122/39	Study area 3	basis	2016/8/1
			basis	2016/8/17
			validation	2016/9/2
MODIS09GA	h27v05	Study area 1	basis	2014/12/5
			predict	2014/12/21
			basis	2015/1/22
	h25v05	Study area 2	basis	2016/3/12
			basis	2016/6/16
			predict	2016/9/20
	h27v06	Study area 3	basis	2016/8/1
			basis	2016/8/17
			predict	2016/9/2

**Table 2:** Landsat8 OLI and MODIS data band comparison table.

Landsat OLI Band	Wavelength (nm)	MODIS Band	Wavelength (nm)
Landsat8 OLI Band2	450–510	MOD09GA Band3	459–479
Landsat8 OLI Band3	530–590	MOD09GA Band4	545–565
Landsat8 OLI Band4	640–670	MOD09GA Band1	620–670
Landsat8 OLI Band5	850–880	MOD09GA Band2	841–876
Landsat8 OLI Band6	1570–1650	MOD09GA Band6	1628–1652
Landsat8 OLI Band7	2110–2290	MOD09GA Band7	2105–2155

**Table 3:** Accuracy assessment results of study area 1.

Band	STARFM		ESTARFM		I-ESTARFM	
	r	RMSE	r	RMSE	r	RMSE
red	0.88530	0.01895	0.90969	0.01812	**0.92767**	**0.01769**
green	0.83601	0.02431	0.93395	0.02283	**0.94859**	**0.02236**
blue	0.89573	0.02800	0.94181	0.02707	**0.95345**	**0.02669**
NIR	0.97119	0.07007	0.97123	0.06893	**0.98162**	**0.06757**
SWIR 1	0.95649	0.04105	0.95477	0.04088	**0.96558**	**0.04005**
SWIR 2	0.94699	0.03801	0.95653	0.03752	**0.96634**	**0.03676**

**Table 4:** Accuracy assessment results of study area 2.

Band	STARFM		ESTARFM		I-ESTARFM	
	r	RMSE	r	RMSE	r	RMSE
red	0.82264	0.04239	0.83780	0.04076	**0.86990**	**0.03919**
green	0.81513	0.04930	0.84313	0.04788	**0.86412**	**0.04519**
blue	0.80664	0.05665	0.81788	0.05521	**0.85654**	**0.05158**
NIR	0.83907	0.06435	0.88136	0.06392	**0.89616**	**0.06287**
SWIR 1	0.72223	0.05237	0.74327	0.05057	**0.76800**	**0.04860**
SWIR 2	0.69582	0.04804	0.73173	0.04585	**0.75836**	**0.04226**

**Table 5:** Accuracy assessment results of study area 3.

Band	STARFM		ESTARFM		I-ESTARFM	
	r	RMSE	r	RMSE	r	RMSE
red	0.80609	0.01420	0.81288	0.01403	**0.85434**	**0.01296**
green	0.83983	0.01709	0.84642	0.01575	**0.90146**	**0.01482**
blue	0.87306	0.02589	0.87751	0.02522	**0.91762**	**0.02361**
NIR	0.90136	0.05373	0.90889	0.05243	**0.93467**	**0.04983**
SWIR 1	0.87413	0.04916	0.88579	0.04853	**0.92770**	**0.04502**
SWIR 2	0.86640	0.03700	0.87866	0.03631	**0.91614**	**0.03540**

**Table 6:** Average computing time of three methods.

Method	STARFM	ESTARFM	I-ESTARFM
Costing time (s)	180	290	320

## References

[1] Adachi M., Ito A., Yonemura S., Takeuchi W. (2017). Estimation of global soil respiration by accounting for land-use changes derived from remote sensing data. J. Environ. Manage..

[2] Senf C., Pflugmacher D., Heurich M., Krueger T. (2017). A Bayesian hierarchical model for estimating spatial and temporal variation in vegetation phenology from Landsat time series. Remote Sens. Environ..

[3] Zhang F., Zhu X., Liu D. (2014). Blending MODIS and Landsat images for urban flood mapping. Int. J. Remote Sens..

[4] González-Sanpedro M., Le Toan T., Moreno J., Kergoat L., Rubio E. (2008). Seasonal variations of leaf area index of agricultural fields retrieved from Landsat data. Remote Sens. Environ..

[5] Gao F., Masek J., Schwaller M., Hall F. (2006). On the blending of the Landsat and MODIS surface reflectance: Predicting daily Landsat surface reflectance. IEEE Trans. Geosci. Remote Sens..

[6] Gao F., Wang P., Masek J. (2013). Integrating remote sensing data from multiple optical sensors for ecological and crop condition monitoring. Remote Sensing and Modeling of Ecosystems for Sustainability X.

[7] Zheng Y., Wu B., Zhang M., Zeng H. (2016). Crop phenology detection using high spatio-temporal resolution data fused from SPOT5 and MODIS products. Sensors.

[8] Hilker T., Wulder M.A., Coops N.C., Linke J., McDermid G., Masek J.G., Gao F., White J.C. (2009). A new data fusion model for high spatial-and temporal-resolution mapping of forest disturbance based on Landsat and MODIS. Remote Sens. Environ..

[9] Roy D.P., Ju J., Lewis P., Schaaf C., Gao F., Hansen M., Lindquist E. (2008). Multi-temporal MODIS–Landsat data fusion for relative radiometric normalization, gap filling, and prediction of Landsat data. Remote Sens. Environ..

[10] Zhu X., Chen J., Gao F., Chen X., Masek J.G. (2010). An enhanced spatial and temporal adaptive reflectance fusion model for complex heterogeneous regions. Remote Sens. Environ..

[11] Kwan C., Zhu X., Gao F., Chou B., Perez D., Li J., Shen Y., Koperski K., Marchisio G. (2018). Assessment of spatiotemporal fusion algorithms for planet and worldview images. Sensors.

[12] Ma J., Zhang W., Marinoni A., Gao L., Zhang B. (2018). Performance assessment of ESTARFM with different similar-pixel identification schemes. J. Appl. Remote Sens..

[13] Fu D., Chen B., Wang J., Zhu X., Hilker T. (2013). An improved image fusion approach based on enhanced spatial and temporal the adaptive reflectance fusion model. Remote Sens..

[14] Zhu X., Helmer E.H., Gao F., Liu D., Chen J., Lefsky M.A. (2016). A flexible spatiotemporal method for fusing satellite images with different resolutions. Remote Sens. Environ..

[15] Knauer K., Gessner U., Fensholt R., Kuenzer C. (2016). An ESTARFM fusion framework for the generation of large-scale time series in cloud-prone and heterogeneous landscapes. Remote Sens..

[16] Gevaert C.M., García-Haro F.J. (2015). A comparison of STARFM and an unmixing-based algorithm for Landsat and MODIS data fusion. Remote Sens. Environ..

[17] Small C. (2004). The Landsat ETM+ spectral mixing space. Remote Sens. Environ..

[18] Ma W.-K., Bioucas-Dias J.M., Chan T.-H., Gillis N., Gader P., Plaza A.J., Ambikapathi A., Chi C.-Y. (2014). A signal processing perspective on hyperspectral unmixing: Insights from remote sensing. IEEE Signal Process Mag..

[19] Song C. (2005). Spectral mixture analysis for subpixel vegetation fractions in the urban environment: How to incorporate endmember variability?. Remote Sens. Environ..

[20] Franke J., Roberts D.A., Halligan K., Menz G. (2009). Hierarchical multiple endmember spectral mixture analysis (MESMA) of hyperspectral imagery for urban environments. Remote Sens. Environ..

[21] Roberts D.A., Gardner M., Church R., Ustin S., Scheer G., Green R. (1998). Mapping chaparral in the Santa Monica Mountains using multiple endmember spectral mixture models. Remote Sens. Environ..

[22] Ridd M.K. (1995). Exploring a VIS (vegetation-impervious surface-soil) model for urban ecosystem analysis through remote sensing: Comparative anatomy for cities. Int. J. Remote Sens..

[23] Aggarwal A., Garg R. (2015). Systematic approach towards extracting endmember spectra from hyperspectral image using PPI and SMACC and its evaluation using spectral library. Appl. Geomatics.

[24] Powell R.L., Roberts D.A., Dennison P.E., Hess L.L. (2007). Sub-pixel mapping of urban land cover using multiple endmember spectral mixture analysis: Manaus, Brazil. Remote Sens. Environ..

[25] Cooley T., Anderson G.P., Felde G.W., Hoke M.L., Ratkowski A., Chetwynd J.H., Gardner J.A., Adler-Golden S., Matthew M.W., Berk A. FLAASH, a MODTRAN4-based atmospheric correction algorithm, its application and validation. Proceedings of the IEEE International Geoscience & Remote Sensing Symposium.

[26] Wayne L. (2016). The IDL Astronomy User’s Library. https://idlastro.gsfc.nasa.gov/ftp/pro/image/correl_optimize.pro.

[27] Cui J., Zhang X., Luo M. (2018). Combining Linear pixel unmixing and STARFM for spatiotemporal fusion of Gaofen-1 wide field of view imagery and MODIS imagery. Remote Sens..

[28] Xie D., Zhang J., Zhu X., Pan Y., Liu H., Yuan Z., Yun Y. (2016). An improved STARFM with help of an unmixing-based method to generate high spatial and temporal resolution remote sensing data in complex heterogeneous regions. Sensors.

[29] Guan Q., Peng X. (2018). High-performance Spatio-temporal Fusion Models for Remote Sensing Images with Graphics Processing Units. AGU Fall Meeting Abstracts.

